# Interpreting a Low Resolution Map of Human U1 snRNP Using Anomalous Scatterers

**DOI:** 10.1016/j.str.2009.05.009

**Published:** 2009-07-15

**Authors:** Chris Oubridge, Daniel A. Pomeranz Krummel, Adelaine K.-W. Leung, Jade Li, Kiyoshi Nagai

**Affiliations:** 1 Medical Research Council Laboratory of Molecular Biology, Hills Road, Cambridge CB2 0QH, England, UK

**Keywords:** PROTEINS

## Abstract

We recently determined the crystal structure of the functional core of human U1 snRNP, consisting of nine proteins and one RNA, based on a 5.5 Å resolution electron density map. At 5–7 Å resolution, α helices and β sheets appear as rods and slabs, respectively, hence it is not possible to determine protein fold de novo. Using inverse beam geometry, accurate anomalous signals were obtained from weakly diffracting and radiation sensitive *P*1 crystals. We were able to locate anomalous scatterers with positional errors below 2 Å. This enabled us not only to place protein domains of known structure accurately into the map but also to trace an extended polypeptide chain, of previously undetermined structure, using selenomethionine derivatives of single methionine mutants spaced along the sequence. This method of Se-Met scanning, in combination with structure prediction, is a powerful tool for building a protein of unknown fold into a low resolution electron density map.

## Introduction

Most proteins in eukaryotic cells exist as components of large protein, RNA-protein, or DNA-protein complexes, which carry out important biological functions in an integrated manner (Gavin et al., 2002). The structure of individual components of these complexes often provides little insight into the structure and mechanism of the assembly of which they are a part. Therefore, the structure of all, or a major part of the assembly must be determined. For example, the structure of individual ribosomal proteins provided little or no insight into protein synthesis or decoding (Ramakrishnan and White, 1998). Therefore, to understand higher order functions of the cell it is important to undertake crystallographic studies of large macromolecular assemblies (Ramakrishnan, 2002; Klein et al., 2004). However, it is very difficult to purify many of these assemblies from natural sources or to assemble them from recombinant components in quantities sufficient for crystallographic studies (Maier et al., 2006; Liu et al., 2006). Crystallization is particularly challenging for large complexes because of sample heterogeneity, which may arise from modification of the sample in vivo, and dissociation of any weakly associated components of the complex as observed with RNA polymerase II (Cramer et al., 2000) and the ribosome (Wimberly et al., 2000). Furthermore, even when these difficulties are overcome, crystals of large complexes often diffract weakly to low resolution and are very susceptible to radiation damage. Fortunately, even low resolution diffraction data (5–7 Å) can provide essential insights into the functions of large macromolecular assemblies, provided one can obtain and interpret an electron density map (Ban et al., 1999; Clemons et al., 1999; Murakami et al., 2002; Bushnell et al., 2004). At such resolutions, α helices appear as tubular density (Muirhead and Perutz, 1963) and β sheets as flat density (Ban et al., 1999; Clemons et al., 1999). It may be possible to place a protein of known structure into the electron density map at this resolution, particularly if its secondary structure is largely α-helical; it is, however, difficult to follow the polypeptide chain of a protein of unknown structure and determine its fold de novo.

We have recently solved the structure, at 5.5 Å resolution, of the functional core of human U1 snRNP, which consists of U1 snRNA, seven Sm proteins, U1-70K, and U1-C (Pomeranz Krummel et al., 2009). In this paper, we show that anomalous peaks from selenium and other heavy atoms could be obtained from highly radiation-sensitive crystals that typically diffracted to ∼6.5 Å. The anomalous peaks were an important aid to fitting known protein folds into the electron density and provided evidence of the quality of the fit. A polypeptide chain of unknown structure was traced on the basis of selenium positions when methionines, which could subsequently be replaced by selenomethionine (SeMet), were introduced by mutagenesis into the sequence at short intervals.

## Results

### Calculation of an Experimental Electron Density Map at 6.5 Å Resolution

The reconstitution and crystallization of U1 snRNP have been described (Muto et al., 2004; Pomeranz Krummel et al., 2009). The functional core of human U1 snRNP consists of U1 snRNA and nine proteins. The crystals grow in *P*1 space group with unit cell parameters *a* = 127 Å, *b* = 128 Å, *c* = 156 Å, α = 96°, β = 107°, and γ = 101° and diffract to ∼6 Å resolution. Self-rotation and self-Patterson analyses suggested four U1 snRNPs in the asymmetric unit (ASU) (data not shown).

**Figure 1:**
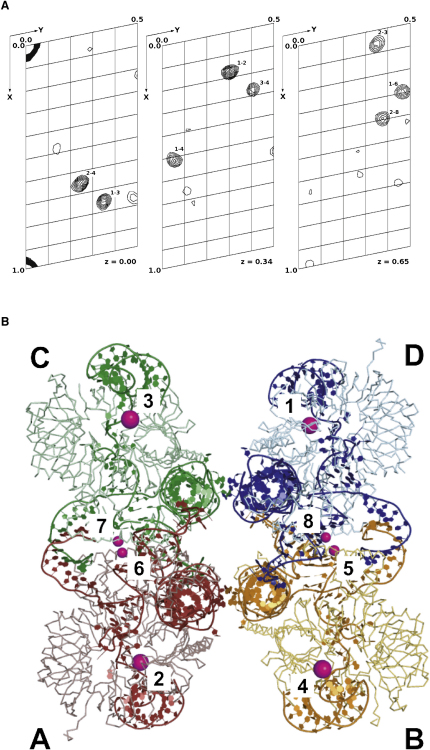
Locating Ta_6_Br_12_ Clusters (A) Three z sections of an anomalous Patterson map calculated from the inflection data of a two wavelength anomalous dispersion experiment. Cross-peaks for all four major sites (origin, 1-2, 1-3, and 1-4) and for one minor site (1-6) can be seen on these sections, as well as a number of other cross-peaks. (B) The major and minor Ta_6_Br_12_ binding sites within the ASU are respectively indicated by large and small magenta spheres and numbered. The four U1 snRNPs in the ASU (particles A, B, C, and D) are colored red, yellow, green, and blue, respectively, with protein shown as ribbon and RNA as cartoon. The Ta_6_Br_12_ clusters bind in cavities between protein and RNA. Major sites lie between SL4 nucleotides 138 and 139 of RNA and the Sm ring residues in the β1-β2 loop of SmB and the β3-β4 loop of SmD1. Minor sites are found between the long α helix of U1-C protein, near Trp41, and the base of SL3 of a NCS-related particle.

A multiwavelength anomalous dispersion data set was collected from a tantalum bromide cluster (Ta_6_Br_12_) derivative (Knäblein et al., 1997) at the Ta L-III edge at two wavelengths: inflection (1.2557 Å) and remote (1.2511 Å). The inflection data were used to calculate an anomalous Patterson map (Figure 1A) and the coordinates of four Ta_6_Br_12_ sites were obtained manually from the cross-peaks. Ta_6_Br_12_ cluster coordinates and occupancies were refined in SHARP (de la Fortelle and Bricogne, 1997). Inspection of residual maps showed four additional minor sites with lower occupancy. Each minor site was 48 Å from a major site, confirming that there were four U1 snRNPs in the ASU, related by noncrystallographic symmetry (NCS), and each bound to two Ta_6_Br_12_ clusters. Spherically averaged form factors of the clusters at 7 Å resolution resulted in higher final phasing power (1.51 versus 1.25), lower Cullis R factor (0.71 versus 0.76), and better overall figures of merit (0.413 versus 0.404) than a single point Gaussian model. Figure 1B shows the packing of four U1 snRNPs in the unit cell and the positions of the four major and four minor Ta sites. The sites were refined with and without coordinate inversion, and the phases were subjected to solvent flipping in Solomon (Abrahams and Leslie, 1996) with a 60% solvent content and extended from 7.5 to 7.0 Å over 11 cycles. The correct hand was identified from better figures of merit for the solvent flattened phases (0.541 versus 0.531) and clear density for A-form RNA in the resulting electron density map.

To improve phasing and to aid location of the Sm proteins, they were all labeled with SeMet then incorporated into U1 snRNP crystals. Se K-edge (λ = 0.9797 Å) data were collected and an anomalous difference map calculated using solvent-flattened Ta_6_Br_12_ phases. The map had 123 peaks greater than 3.0 SD (σ) above background (data not shown).

To further improve phasing and to locate the Zn-finger protein U1-C, an anomalous map was calculated from native data collected at the zinc K edge (λ = 1.2827 Å). The map showed four peaks greater than 7.8 σ with the next strongest being 4.4 σ. The four strongest peaks correspond to zinc atoms in U1-C protein, in agreement with there being four U1 snRNP complexes in the ASU.

The coordinates of Se atoms, Zn atoms, and Ta_6_Br_12_ clusters were refined together against their respective data sets in SHARP (de la Fortelle and Bricogne, 1997). Occupancy and position were refined for all sites, except for one Ta_6_Br_12_ site, which was fixed at the origin. B factors were fixed at 200.0 Å^2^. The phases were improved by solvent flipping in Solomon with a 60% solvent content, extended from 7.0 Å to 6.5 Å over 11 cycles and had final overall figures of merit of 0.652.

### Model Building

#### RNA

The resulting electron density map was of high quality such that RNA helices, with shallow minor grooves and deep major grooves, were readily discernible. It was not always possible to fit long fragments of A-form RNA into extended regions of helical density, they were therefore initially built from short fragments of idealized A-form RNA and manually rebuilt around the junctions to improve stereochemistry. An NMR structure of a kissing loop RNA (Kim and Tinoco, 2000; PDB code 1f5u) was built into the electron density at the apical part of stem loop 2 (SL2). This domain had been introduced into the RNA to promote crystal lattice interactions (Pomeranz Krummel et al., 2009), which are seen within the ASU between U1 snRNP complexes A and B and complexes C and D (Figure 1B).

#### Sm Proteins

The structure of the U4 snRNP core domain, an assembly of seven Sm proteins and a 68 nucleotide RNA, was solved to 3.6 Å resolution independently (A.K.W.L., J.L., and K.N., unpublished data) and four of the protein structures have been determined as heterodimers of SmD1D2 and SmBD3 (Kambach et al., 1999). One to one correspondence was found between the majority of our selenium anomalous peaks from SeMet-labeled Sm proteins and the methionine residues in the U4 core domain whereas some anomalous peaks are composite peaks arising from two or more selenium atoms. The U4 core domain structure (A.K.W.L., J.L., and K.N., unpublished data) was initially fitted into the U1 snRNP map by superposition of the U4 core methionine sulfur positions onto the corresponding refined Se sites. In the U1 snRNP map the N-terminal helices of the Sm fold were fitted into tubular densities but the electron density for the β sheet in some subunits was discontinuous.

#### U1-C

The lowest energy NMR structure of the Zn-finger domain of U1-C (Muto et al., 2004; PDB code 1uw2) was placed at the Zn anomalous peak positions based on the rod-like density of the α-helical region. A long section of α-helical density from U1 snRNP complex A extends toward SL3 of complex C and vice versa, and complexes B and D show the same relationship. This long α-helix of U1-C shows that helices B and C, which form a turn with a flexible loop in isolated U1-C in the solution structure (Muto et al., 2004), form a continuous helix in the U1 snRNP crystal. In order to ensure correct orientation of the Zn-finger domain a native crystal was treated with ethyl mercury thiosalicylate (EMTS). An anomalous peak of mercury (Hg) bound to Cys-25 adjacent to Zn-coordinating His-24 was used to place the Zn-finger domain more precisely. An additional Hg anomalous peak from a single Cys mutant (Q39C) was used to orient the C-terminal helix and place it in the correct register.

#### U1-70K

**Figure 2:**
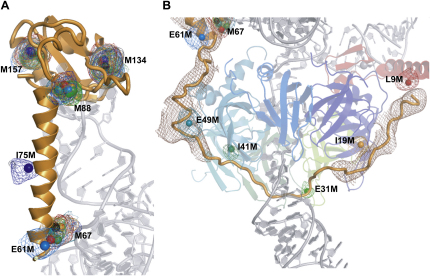
Overlay of Selenium Peaks from Multiple Crystals Containing U1-70K SeMet Mutant Protein (A) U1-70K residues 61–180 are shown as orange cartoon, with part of U1 snRNA, including SL1, shown in light gray. The selenium peak coordinates from anomalous maps of the eight U1-70K mutants are marked by colored spheres. The selenium anomalous maps are shown, all contoured at 3.5 σ and colored to match the spheres. Sphere diameter is ∼2 Å. As well as the four natural methionines (67, 88, 134, and 157), which have corresponding peaks in all the mutants, two of the mutant site peaks (E61M and I75M) are also shown. The colors are: wild-type, black; L9M, red; I19M, orange; E31M, light green; I41M, dark green; I49M, cyan; E61M, blue; I75M, dark blue. (B) The path of the extended N terminus of U1-70K. Electron density attributed to U1-70K is shown in brown and contoured at 1 σ. Where density is absent, approximately between residues 24 and 45, a plausible path for the peptide is indicated based on the selenium positions of E31M and I41M. Selenium peaks and anomalous maps are as for (A). Near the selenium site of L9M, U1-70K is seen to interact with U1-C, which is red.

The RNA binding domain (RBD) of U1-70K was known to bind to SL1 (Patton and Pederson, 1988; Query et al., 1989) and a large globule of electron density was seen in the SL1 loop region. The initial map did not permit unambiguous fitting of the RBD, so U1-70K was labeled with SeMet and reconstituted into U1 snRNP. The resulting crystal gave four Se anomalous peaks (above 4.0 σ) per U1 particle. Two of the peaks, corresponding to Met-134 and Met-157, lie within the RBD (Figure 2A). The RBD was homology modeled from the N-terminal RBD of U1A (Nagai et al., 1990; Oubridge et al., 1994) and placed using these peaks along with the rod-like density of its two α helices. The loop region of SL1 was built in such a way that G28 and U30 are in close proximities of Tyr112 and Leu175, which were assumed from cross-linking data (Urlaub et al., 2000). The remaining two anomalous peaks were found in the long rod-like density adjacent to SL1. This region was predicted to be α-helical and was modeled as such between residues 63 and 89. Further support for this model comes from the observation that many of the basic residues of the helix are close to the phosphate backbone of SL1, favoring electrostatic interactions (Pomeranz Krummel et al., 2009).

The location and the structure of the N-terminal 62 residues of U1-70K were unknown, so we made several single methionine mutants in this region to locate these residues from the position of anomalous peaks when U1 snRNP was reconstituted with their SeMet derivative. The amino acids chosen for substitution with methionine had long and/or hydrophobic side chains, in order to minimize structural changes. Basic side chains were avoided because their mutation could disrupt RNA binding. Substitutions were made at approximately ten residue intervals to facilitate chain tracing. Data from crystals grown with SeMet derivatives of these mutants gave selenium anomalous difference peaks that revealed an extended polypeptide from residue 9 to 63 (Figure 2B). The polypeptide interacts with U1-C protein and traces a path around the Sm protein ring to the beginning of the α helix adjacent to SL1. Figure 2A shows an overlay of Se peaks for the natural methionine residues of U1-70K that are present in all these crystals. The selenium anomalous peaks of these residues are clustered with rms < 1.9 Å, except for Met67 in complex B (rms = 2.5 Å). The observed scatter was within the expected positional errors at this resolution.

### Phase Extension to 5.5 Å: Multi-NCS, Multi-Crystal Averaging

An EMTS-soaked crystal of U1 snRNP, containing the Q39C mutant of U1-C, diffracted to 5.5 Å, compared with 6.5 Å for the Sm protein SeMet derivative crystal from which, along with the Ta_6_Br_12_ derivative and zinc edge data, the original experimental map was calculated. The unit cell of this crystal had a *c* axis 3.7 Å shorter than the mean of the other crystals' *c* (152.0 Å compared to average of 155.7 ± 0.7 Å; see Table S1 in Pomeranz Krummel et al. (2009) for crystal statistics). The EMTS-soaked U1-C Q39C crystal also exhibited lower mosaic spread than the other crystals. Because the crystal was not isomorphous with those used to calculate the original maps, we used multi-NCS, multi-crystal averaging to take advantage of both the 4-fold redundancy of U1 particles in the ASU and the superior diffraction of the U1-C Q39C-EMTS.

**Figure 3:**
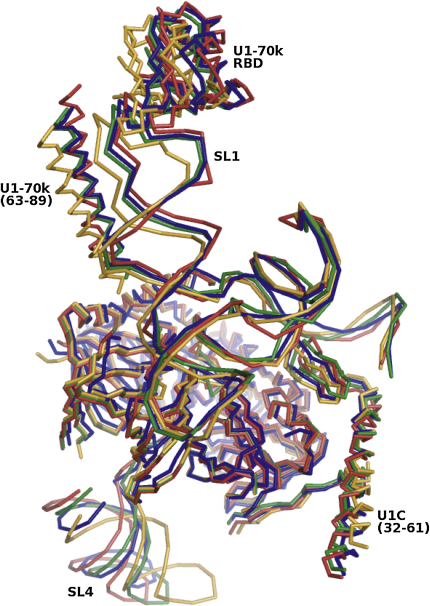
Superposition of NCS-Related Particles The four particles in the ASU were superimposed in O (Jones et al., 1991) using the Cα atoms of Sm protein residues within the Sm fold, U1-70K residues 9–23, and the P atoms of U1 snRNA nucleotides 1–16 and 48–134. Particles B, C, and D were transformed onto particle A with rms of 1.75 Å, 2.03 Å, and 1.97 Å for those Cα and P atoms, respectively. The particles are shown as ribbons with A, B, C, and D in blue, green, yellow, and red, respectively. The substructures that varied in their relative orientations between particles, and which were treated as separate domains in averaging, are indicated. SL1, U1-70K RBD, and U1-70K helix (63–89) were combined and treated as a single domain. SL4 and U1-C C-terminal helix (32–61) were both treated as separate domains.

Attempts to superimpose the four U1 snRNP complexes in the ASU (Figure 3) showed that there were small but significant differences between the positions of the following substructures: (1) RNA residues 1–16 and 48–134, U1-C residues 4–31, U1-70K residues 9–23, and proteins Sm-D3, B, D1, D2, F, E, and G; (2) SL1, U1-70K RBD, and residues 63–89; (3) U1-C residues 32–61; (4) SL4. NCS transformation matrices between the substructures in the four U1 snRNP particles within the ASU were generated in O (Jones et al., 1991). Masks were created for each of these substructures using the program NCSMASK (CCP4, 1994). The masks, the solvent-flattened phases at 6.5 Å, the SeMet Sm core data, and the U1-C(Q39C)-EMTS crystal data were used for multidomain, multi-crystal averaging in the program DMMULTI (Cowtan et al., 2001). This resulted in phases with mean figures of merit of 0.623 to 5.5 Å, which compares well with a value of 0.652 to 6.5 Å for the phases used to calculate the experimental map. The resulting 5.5 Å map was clearly of higher quality than the original map: density for β sheets of the Sm proteins became continuous and some RNA density revealed phosphate group bumps. This enabled the U1 snRNP model to be built with more accuracy and certainty than had been possible with the original map.

### Accuracy of Sm Protein Selenium Positions at 5.5 Å Resolution

**Figure 4:**
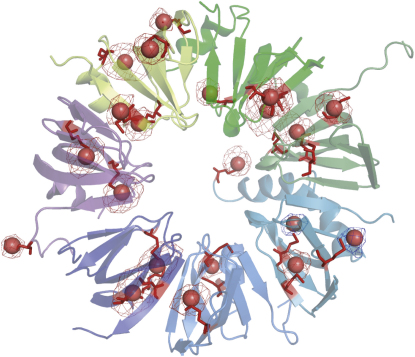
Positioning of Sm Proteins from U4 Core Structure onto U1 snRNP Selenium Peak Positions The anomalous difference map was calculated using data from U1 snRNP crystals that contained SeMet-labeled Sm proteins. The anomalous peaks are contoured at 3σ (red mesh) and 2σ (blue mesh). The selenium anomalous peak positions are shown as red spheres. The Sm protein ring of the U4 core domain is shown as a cartoon with methionine residues colored red.

We have shown that the use of heavy atom landmarks is a powerful method for the interpretation of low-resolution structure. Since NCS and multi-crystal averaging had improved the quality of the map considerably, we used the improved phases to recalculate anomalous difference maps of the U1 snRNP crystals containing the Se-Met derivative of Sm proteins (Figure 4). The seven Sm proteins contain a total of 25 methionines within the Sm fold, most of which we expected to be ordered in the crystal. We observed 20 anomalous peaks above background (for particle A), which have been assigned to single methionines. All but one (Met11 of SmD2) are within the Sm fold. Furthermore, two of the peaks appear to be composite peaks arising from groups of three (SmB: Met-9, Met-38, and Met-80) and two (SmE: Met-78 and SmF: Met-40) methionines. We assume that the selenium atoms of these residues are close enough in space for the peaks to merge at 6.0 Å resolution. Two of the methionines in this particle have no corresponding selenium signal (SmD1: Met-36 and SmF: Met-27), presumably because the methionine side chains are disordered in our crystals. The assignment of methionines to selenium anomalous peaks is similar for the other three particles. The mean rmsd of the overlays between anomalous peak positions and the methionine sulfur positions in the U4 core domain for the four U1 particles is 2.22 ± 0.08 Å, excluding any composite peaks. The deviations in position arise from several sources: coordinate error of the U1 selenium peaks, coordinate error of the U4 core structure, and genuine differences between the U1 and U4 structures. The latter may arise from the Sm proteins being bound to distinct RNAs, making different crystal contacts and the crystals being grown under different conditions (A.K.W.L., J.L., and K.N., unpublished data). A peak found outside the Sm fold was connected to SmD2 by a kinked rod-like density suggesting α helices. This peak is attributed to Met11 of SmD2. If α helices are built into the density, extending the N-terminal region from that seen in the SmD1D2 heterodimer (Kambach et al., 1999), then Met11 can account for the peak. The U1 map reveals other regions that have shifted relative to the U4 core structure. Many of these are at the N and C termini, outside the canonical Sm fold. One of the most notable changes is for SmF protein residues 6 to 15. There are also conformational differences in some loop regions within the Sm fold, such as SmD3 residues 49 to 55 between strands β3 and β4 and SmF residues 46 to 56, which are explainable by interaction with the N-terminal peptide of U1-70K and with a neighboring complex, respectively. Overall, the structures appear to be the same within the canonical Sm fold.

### Accuracy of U1-70K Selenium Positions at 5.5 Å Resolution

The U1-70K protein mutants provide useful information regarding the accuracy of atom positions determined by this method. We were able to trace the extended N-terminal region of U1-70K by SeMet labeling. What is the accuracy of selenium positions determined by this method? Table 1 summarizes the data statistics of the crystals containing these Se-Met-labeled proteins. The means and SD are given for each of the cell dimensions. The largest variations are seen with the *c* axis, which has an SD of 0.36 Å, the others have values of 0.12–0.17 Å.

All the mutants contain common natural methionine residues: two in the RBD (Met-134 and Met-157) and two in the α-helical region (Met-67 and Met-88). Figure 2A shows the positions of the anomalous peaks together with the model of the RBD and the α helix in particle A. The sigma values of these sites are compared in Table 2. Most sites are above 4σ but in 5 out of 140 cases no significant peaks were found, even though strong peaks were found in other particles. It is unclear why anomalous peaks were not found but it is unlikely to be due to disorder of the sites because the methionines would be located in similar environments in all the particles in the ASU. Table 3 shows an SD from the mean of the anomalous peak positions for each site when the sites from the four particles are superimposed. Table 4 shows SD from the mean for natural sites within each particle. These are similar to the values from interparticle superposition, except for Met-67 (Table 3), and this shows that differences between the particles do not make a major contribution to the SD values in Table 3. Met-67 side chain appears to be more mobile than the other sites in particle B. We can offer no explanation for this, but an inspection of Tables 3 and 4 shows that it is an outlier. With the exception of Met-67 the values are below 2 Å; therefore, at 5.5 Å resolution the position of an anomalous scatterer can be determined to within 2 Å when crystals show only small variations in cell dimension (Table 1).

The Se atoms of the mutant sites all gave significant anomalous peaks, except for the E49M site in particle C (Table 2). We also compared the positions of the mutant Se-Met sites by overlaying the U1-70K mutant and wild-type anomalous peak positions of the four particles. The mutant Se anomalous peaks all superimposed with SD values of less than 2 Å (Table 3).

## Discussion

We obtained our initial electron density map using a tantalum bromide derivative. Data were collected in inverse beam mode with 1° wedges to measure anomalous signals accurately (see Data Collection in Experimental Procedures). This strategy resulted in a strong anomalous signal. Surprisingly, when the wedge size was increased to 5°, we obtained a much weaker anomalous signal from several different crystals, as judged by correlation coefficients within (I^+^) and (I^−^), measured in SCALA (CCP4, 1994; Evans, 2006), and the lack of significant peaks in the anomalous difference map. We attribute this to rapid decay of the anomalous signal during data collection. For the radiation-sensitive *P*1 crystal of U1 snRNP the use of inverse beam mode was absolutely essential. We showed that the position of heavy atom scatterers such as Se, Zn, and Hg could be determined with a positional error of <2 Å at 5.5 Å resolution (Tables 3 and 4). We were able to place the 3.6 Å structure of the U4 core domain by least-squares fit of methionine sulfur atoms onto anomalous peak positions in the U1 map with an rmsd of <2.4 Å. The agreement between the electron density and the U4 structure is excellent within the Sm fold but the map also indicates some differences between the structures in regions for which flexibility would be expected, such as loops and N- and C-terminal extensions beyond the Sm fold. It is not difficult to place a protein of known structure into an electron density map at this resolution and any ambiguities may be resolved by judicious use of heavy atom markers. We were able to trace the extended chain of the N terminus of U1-70K using Se-Met scanning. Hence, in principle it will be possible to trace the chains of other proteins of unknown structure with the same method at resolutions as low as 6.5 Å. Residues 63–88 of U1-70K were predicted to form an α helix using Jpred3 (Cole et al., 2008). When an idealized α helix was fitted into a tubular density, the distance between the two methionines (Met-67 and Met-88) and their relative orientations with respect to the helical axis were consistent with the model. When Ile75 was mutated to methionine, its position and orientation were also consistent with the model. This shows that α helices can be placed at correct position and orientation with a positional error of 2 Å at this resolution, providing important information that can be tested experimentally. In contrast with α helices, extended polypeptide strands are not always resolved at this resolution. Introduction of Se-Met at key positions provided landmarks to reveal the path of a strand of polypeptide, even where protein density was not observed in the experimental maps. In combination with secondary structure prediction, this method can elucidate more significant structural information from diffraction data at moderate resolution than can the electron density alone.

## Experimental Procedures

### SeMet Derivatives

All SeMet-labeled proteins were expressed in a methionine auxotrophic strain of *E. coli* using the method as described in Ramakrishnan et al. (1993). U1-70K was expressed as an N-terminal fusion with thioredoxin followed by a (His)_6_ tag and a TEV protease cleavage site (Ignjatovic, 1999). The protein was purified on a Ni-NTA agarose column followed by proteolysis with TEV protease and a second Ni-NTA agarose column. Sm proteins were purified as previously described (Kambach et al., 1999). Reconstitution and crystallization of U1 snRNP have been described previously (Muto et al., 2004; Pomeranz Krummel et al., 2009).

### Heavy Atom Derivatization

A tantalum bromide cluster (Ta_6_Br_12_; Knäblein et al., 1997) derivative was prepared by directly adding a few grains of the dark green solid into crystallization drops and incubating for 6–8 hr at 4°C. The grains dissolved and the cluster compound soaked into the crystals. After incubation the crystals turned a darker shade of green than the surrounding mother liquor and were cryo-cooled as described below, without back soaking.

Crystals were grown by hanging drop vapor diffusion from U1 snRNP reconstituted with the Q39C mutant of U1-C_(1-77)_ protein (Pomeranz Krummel et al., 2009). The crystal mother liquor was exchanged with EMTS dissolved in the well buffer to a final concentration of 1 mM and incubated at 4°C for 20 hr. The crystal was back soaked in well buffer and cryo-cooled as described below.

### Cryo-Cooling

Crystals were grown in the presence of 38%–40% 2-methyl-2,4-pentanediol and did not require further cryo-protection. Cryo-cooling was carried out by lifting crystals from the mother liquor on loops at 4°C and flash cooling in the gas jet of a cryostream (Oxford Cryosystems) at 100 K. All these steps were done at 4°C. Freezing in a cryostream consistently reduced the mosaicity of the crystals, as opposed to plunging the crystal directly into liquid nitrogen.

### Data Collection

Diffraction data were collected on a Mar225 CCD detector at Swiss Light Source X06SA and X10SA beam lines with the X-rays focused on the detector. In order to minimize the effect of radiation damage inverse beam mode was used; i.e., after every 1° oscillation the crystal was rotated 180° such that Friedel pairs were measured nearly simultaneously. Data were integrated with MOSFLM (Leslie, 2006) and scaled with SCALA (CCP4, 1994; Evans, 2006). Phasing has been described in the text as well as in Pomeranz Krummel et al. (2009).

Figures were prepared using PyMol (DeLano, 2002) except for Figure 1A, which was made in Mapslicer (CCP4, 1994).

## Figures and Tables

**Table 1:** The Cell Parameters and Data Statistics of the U1 snRNP Crystals with the SeMet Derivatives of Different U1-70k Mutants

SeMet									
U1-70k			Cell dimensions				Wavelength	Resolution	Observations
Data set	a (Å)	b (Å)	c (Å)	α(°)	β (°)	γ (°)	(Å)	(Å) (Outer Bin)	
Wild-type	126.7	127.2	154.3	96.1	106.6	101.3	0.9787	100.0–7.5 (7.9–7.5)	130,493 (19,872)
L9M	127.1	127.5	154.5	95.7	106.5	101.6	0.9790	70.0–6.6 (7.0–6.6)	69,457 (10,234)
I19M	127.0	127.4	154.1	95.9	106.6	101.5	0.9789	100.0–6.3 (6.6–6.3)	189,353 (27,707)
E31M	127.0	127.5	154.8	96.1	106.4	101.2	0.9791	68.2–6.7 (7.1–6.7)	123,897 (18,401)
I41M	127.0	127.6	154.7	96.2	106.2	101.3	0.9792	73.1–6.7 (7.1–6.7)	132,275 (19,611)
E49M	127.1	127.7	153.8	96.0	106.2	101.6	0.9792	72.7–6.5 (6.9–6.5)	145,565 (21,349)
E61M	126.9	127.4	154.7	96.2	106.6	101.3	0.9792	73.3–6.7 (7.1–6.7)	131,734 (19,590)
I75M	127.0	127.5	154.9	96.2	106.6	101.3	0.9792	70.0–6.7 (7.1–6.7)	132,203 (19,768)
Mean	126.98	127.48	154.48	96.05	106.46	101.39			
SD	0.12	0.14	0.36	0.17	0.17	0.15			

SeMet							
U1-70k		Anomalous		Anomalous			
Data set	Multiplicity	Multiplicity	Completeness	Completeness	R_merge_^a^	R_p.i.m._^b^	Mn([I]/sd[I])
Wild-type	13.0 (13.3)	6.5 (6.7)	88.2 (89.8)	86.7 (88.1)	21.4 (96.5)	6.8 (27.7)	43.7 (6.4)
L9M	4.2 (4.2)	2.1 (2.1)	98.7 (98.9)	98.3 (98.7)	4.5 (50.2)	3.0 (33.7)	15.5 (2.3)
I19M	9.9 (9.9)	4.9 (5.0)	99.1 (99.2)	98.9 (98.9)	6.7 (113.7)	2.4 (37.6)	9.3 (2.7)
E31M	7.8 (7.8)	3.9 (3.9)	98.6 (98.6)	98.2 (98.3)	10.3 (82.5)	4.2 (33.5)	15.3 (2.2)
I41M	8.3 (8.3)	4.1 (4.2)	98.7 (98.9)	98.4 (98.8)	6.3 (90.3)	2.5 (35.2)	17.9 (2.5)
E49M	8.3 (8.4)	4.2 (4.2)	99.1 (99.2)	98.7 (99.2)	11.4 (63.5)	4.4 (24.4)	15.5 (3.2)
E61M	8.3 (8.3)	4.1 (4.2)	98.7 (98.7)	98.4 (98.7)	7.4 (107.8)	2.9 (42.3)	16.3 (1.9)
I75M	8.3 (8.3)	4.1 (4.2)	98.8 (98.9)	98.4 (98.7)	11.1 (89.8)	4.3 (34.8)	14.9 (2.0)

a Merging *R* factor $$R_{merge}=\sum_{hkl}\sum_{i}|I_{i}\left(hkl\right)-\bar{I\left(hkl\right)}|/\sum_{hkl}\sum_{i}I_{i}\left(hkl\right)$$

b Precision-indicating merging *R* factor $R_{\mathrm{p.i.m.}}=\sum_{hkl}{\left[1/\left(N-1\right)\right]}^{1/2}\sum_{i}|I_{i}\left(hkl\right)-\bar{I\left(hkl\right)}|/\sum_{hkl}\sum_{i}I_{i}\left(hkl\right)$

**Table 2:** The Peak Sizes of the Anomalous Peaks of Natural Methionines in U1-70k and of the Mutant Sites for Each of the Molecules in the ASU

	σ, M67	σ, M88	σ, M134	σ, M157	σ, MT site
L9M, MolA	4.3	5.7	7.9	10.7	6.3
L9M, MolB	4.9	4.7	6.8	10.9	7.5
L9M, MolC	6.4	4.7	8.2	7.6	8.0
L9M, MolD	4.7	5.8	8.0	7.6	8.2
I19M, MolA	5.7	6.9	6.9	8.8	7.4
I19M, MolB	5.0	4.7	8.4	10.3	7.0
I19M, MolC	5.0	5.3	5.8	7.4	6.5
I19M, MolD	5.9	7.7	8.3	8.3	6.4
E31M, MolA	5.5	4.8	6.5	5.9	5.0
E31M, MolB	-	4.9	5.9	8.7	4.8
E31M, MolC	4.2	8.3	6.9	4.5	6.3
E31M, MolD	6.2	4.1	4.9	6.4	5.9
I41M, MolA	5.1	4.7	5.7	6.7	4.7
I41M, MolB	4.8	4.7	4.2	6.4	6.2
I41M, MolC	4.0	4.7	6.4	6.6	4.2
I41M, MolD	4.9	4.8	6.0	6.8	7.9
E49M, MolA	5.8	5.9	6.4	8.7	7.6
E49M, MolB	6.2	4.7	6.9	6.5	4.3
E49M, MolC	4.9	5.3	8.5	9.7	-
E49M, MolD	4.4	4.8	8.1	12.8	8.8
E61M, MolA	-	5.7	7.7	7.7	5.2
E61M, MolB	4.2	4.7	6.2	10.9	6.7
E61M, MolC	4.9	5.0	7.0	4.0	5.3
E61M, MolD	4.7	4.7	4.6	-	5.1
I75M, MolA	-	5.0	7.1	5.5	4.8
I75M, MolB	4.8	4.4	5.7	6.1	4.5
I75M, MolC	4.2	3.8	7.1	6.8	4.9
I75M, MolD	5.5	5.1	5.9	5.9	5.4

**Table 3:** Overall Rmsd for Mutant and Native Methionine Sites from all Four Particles

Residue/Site	Number of Atoms	SD from the Mean (Å)
L9M	4	1.4
I19M	4	1.2
E31M	4	1.3
I41M	4	1.9
I49M	3	1.9
E61M	4	1.4
I75M	4	1.6
M67	27	2.5
M88	29	1.6
M134	32	1.1
M157	31	1.2

Three matrices were generated for the superposition of U1-70k selenium sites from U1 snRNP complexes B, C, and D onto the U1-70k selenium sites of complex A, using the program O (Jones et al., 1991). An SD from the mean was then calculated for each site from the transformed coordinates. $SD=\sqrt{\frac{1}{n}\cdot \sum_{i=1}^{n}{\left(\bar{x}-x_{i}\right)}^{2}+{\left(\bar{y}-y_{i}\right)}^{2}+{\left(\bar{z}-z_{i}\right)}^{2}}$

**Table 4:** Intraparticle SD Values for Native Methionine Sites, Including Wild-Type SeMet U1-70k

Site	Particle in ASU	Number of Atoms	SD from Mean (Å)	Mean SD for Site
Se-67	A	5	1.7	1.9
	B	7	2.7	
	C	7	1.9	
	D	8	1.4	
Se-88	A	7	1.1	1.6
	B	7	1.7	
	C	8	1.9	
	D	7	1.6	
Se-134	A	8	1.1	1.0
	B	8	1.0	
	C	8	1.0	
	D	8	1.0	
Se-157	A	8	1.3	1.2
	B	8	1.2	
	C	8	1.1	
	D	7	1.1	

The SD from the mean was calculated from the coordinates of seleniums of wild-type seleniums within each particle, as formulated in Table 3.

## Supplemental Data

Movie S1. A Cartoon Representation of the U1 snRNP, Rocking by ±30 Degrees about theY-Axis The RNA (U1 snRNA) is in gray, U1-70k protein in orange, U1-C in red and the Sm proteins D3, B, D1, D2, F, E and G are colored dark-blue, blue, sky-blue, teal, cyan, dark-green and light green, respectively. The seven Sm proteins are bound in a ring around the conserved Sm site of the RNA. The RNA binding domain and flanking helix of U1-70k protein are bound to RNA stem-loop 1 (SL1), on the top left. SL2 emerges from the center of the particle and points toward the viewer. SL1 and SL2 are co-axially stacked and lie approximately at right angles to SL3, which points upward from the center of the particle. SL3 is co-axially stacked with a short region of RNA duplex, helix H. SL1, SL2, SL3 and helix H together form a four-way junction. SL4 points down to the right from below the ring of Sm proteins. SL2 is truncated and replaced with a kissing loop sequence to promote a crystal lattice interaction. The extended N-terminal polypeptide of the U1-70k protein wraps around the outside of the ring of Sm proteins. This part of the protein was traced de novo at 6.5 Å resolution by introducing methionine residues into U1-70k and crystallizing the particle with selenomethionine derivatives of the mutant proteins. The selenium atom positions are shown as colored spheres.The N terminus of U1-70k is required for binding of the U1-C protein, which is bound between the Sm-D3 protein, the N terminus of U1-70k protein and the 5′-end of the U1 snRNA. This part of the RNA is bound to an equivalent RNA from an NCS-related particle in a way that we think mimics U1 snRNP's binding to the 5′ splice site. U1-C binds to the RNA duplex formed by this interaction and may therefore play a role in stabilizing 5′ splice site binding.
